# Safety of Hypnotism as a Remedy

**Published:** 1891-07

**Authors:** 


ARTICLE VIII.

### SAFETY OF HYPNOTISM AS A REMEDY.

So much has been written lately of the dangers of hypnotism that it is interesting to note a very interesting and remarkable article on the therapeutic use of hypnotism, by Dr. Hamilton Osgood in the *Boston Medical and Surgical Journal*, March 26, 1891. In concluding, Dr. Osgood says: I wish again to allude to the feeling that hypnotism is dangerous. If it be applied with the necessary technical understanding and the proper suggestions, the causation of hallucinations being avoided, and if the patient be treated with the gentleness and consideration which is always shown a patient under any other circumstances, *I distinctly deny that danger attends the use of hypnotism.* I do not speak from hearsay, but from a moderately large experience. It is easy to see how harm could arise where a susceptible patient is used as a plaything for the creation of hallucinations, day in and day out, at the sweet will of medical students and of physicians indifferent to all else than their own amusement, indulgence of curiosity or a desire to watch effects. Charcot's patients are trained; *strained* one may say, to the highest degree and by startling and exhaustive methods. It is no wonder that he sees nervous disturbance among them, and that they became mere puppets. Charcot, therefore, declares that only hysterical subjects or persons of what he calls "a peculiar nervous make-up," can be hypnotized, and that hypnotism will create hysteria. This is curiously refuted by all the statistics published by men who hypnotize according to the Nancy methods, men whose united testimony refers to hundreds of patients to Charcot's one. This is my experience, and further, while the larger number of my patients have been non-hysterical, among them strong men, those who were hysterical, and have responded to the treatment, have become free from the nervous ailment.

Is there no argument in Bernheim's decided assertion, after more than nine years of large experience, that he never has seen one case in which harm of any sort has resulted? He shows how harm could be caused, but also that it is wholly unnecessary. I can show that harm has been caused by the use of baths; but does one stop bathing? Liebeault has used hypnotic suggestions during thirty years of large practice, and *he* pronounces its proper application harmless. It is singular that mere prejudice strikes stronger roots in some minds than simple truth. The fact is, however, that the laity is more independent of the opinion of the physician than once was the case. People will decide for themselves, and the very results of hypnotic suggestion will be sufficient proof.

A well-known physician, who is a good hater of hypnotism, but who, of course, has not used it himself, else he would have another opinion, said to a relative of a patient whose case I have mentioned, that hypnotism was hurtful. "Yes," was replied, "but there is my cousin, who for twenty years was constantly intemperate and now cannot be made to touch liquor, and his health has greatly improved. This was the result of hypnotism. That is proof enough for me."

Can any fair-minded men fail to see the great value of the treatment in view of such results? One can merely quietly continue the use of a remedy which, in many senses, nothing can replace, and let prejudice take care of itself. No physician, who practically has followed the methods of the Nancy School, confining suggestions to the functions of the organism, will admit that he has never seen a result in any sense injurious to the patient. Consider the vast number of patients which this statement in-includes!

One argument against the use of hypnotism I cannot leave unmentioned. It is, that in using it a physician, simply because this thing has in the past been largely in the hands of quacks, might injure his practice or hurt his standing. This needs only one word of reply: To put it midly, it is the argument of a man without courage. Nevertheless, the timid physician, who *believes* in hypnotism, doubtless finds reason to be thankful for the fact that to-day hypnotic suggestion stands firmly upon scientific ground, and that it is practiced abroad by men whose judgment is ripe, and whose medical attainments occupy the first rank. Personally, I should feel that I were doing wrong, and were disobeying duty, if I were unwilling to apply hypnotic suggestion wherever I saw it were likely to aid the patient. And this is the outcome of my experience in this branch of therapeutics.—*Medical and Surgical Reporter.*

